# Analytical dataset to determine squeezing potential of deep tunnels

**DOI:** 10.1016/j.dib.2025.111808

**Published:** 2025-06-18

**Authors:** Ferlien Margareth Mareyke Mombilia, Simon Heru Prassetyo, Yudhidya Wicaksana, Ridho Kresna Wattimena, Anatasya Claresta

**Affiliations:** Mining Engineering Study Program, Institut Teknologi Bandung, Bandung, Indonesia

**Keywords:** Tunnel strain, Stability factor, Support pressure, Ground reaction curve, Squeezing, Deep tunnel

## Abstract

This article presents a dataset that consists of historical strain data from deep tunnel construction and an analytical study that uses the ground reaction curve (GRC) to calculate the support pressure required to lower the risk of squeezing potential. In deep tunnel excavation, high in-situ stress conditions can cause an instability phenomenon known as squeezing. Historical tunnel strain data are classified into categories based on the percentage of tunnel convergence observed. Furthermore, 480 ground reaction curves (GRC) were developed and analyzed: 5280 calculations were performed with tunnel radii of 3, 4, and 5 m, depths ranging from 100 to 1000 m (in increments of 100 m), uniaxial compressive strength *(σ_ci_*) values of 30 and 50 MPa, and Geological Strength Index (*GSI*) values from 20 to 90 (in increments of 10). Three primary factors were used to estimate support capacity: the reduction of internal pressure relative to in-situ stress (*p_i_/p_o_*), strain (*%ε*), and the stability factor (*σ_cm_/p_o_*). Strain (*%ε*) and the stability factor (*σ_cm_/p_o_*) were classified based on the resulting *p_i_/p_o_* (0–0.9). A graph was obtained to determine the minimum *p_i_/p_o_* that reflects all rock mass class conditions in deep tunnel construction in an effort to avoid squeezing at specific limits.

## Graphical abstract

**Figure:**
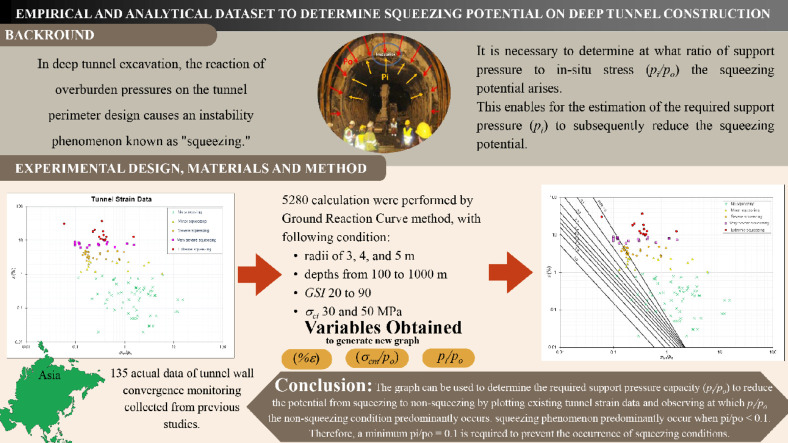

Specifications Table

**Table:** 

Subject	Engineering & Materials science
Specific subject area	Calculation of support capacity in deep tunnel construction to reduce squeezing potential
Type of data	Table, raw, analyzed, Excel file, TIFF images
Data collection	A total 135 data points of tunnel strains were gathered from various literature sources, as indicated in the Excel table (file Dataset GRC.xlsx) and in the reference list. These data present the convergence on tunnel walls, obtained from actual field measurements. No previous study has collected tunnel convergence data in quantities equal to or exceeding those in this research. These data are highly valuable for analyzing the rock type factors that cause squeezing, determining the percentage of convergence that causes squeezing, and identifying the minimum support capacity based on the *p_i_/p_o_* ratio to prevent squeezing occurrences.
Data source location	Tunnel strain data in this study are secondary data collected from various literature sources: 94 % of the points are from tunnel construction projects in Nepal, India, and Turkey, while the remaining 6 % come from tunnels in France and China. The literature sources from which the strain data were obtained are listed below: Barla G, Tunnelling in squeezing rock condition, Proceedings of ROCKSITE-99 India, (1999) Dehkordi MS, Shahriar K, Moarefvand P, Gharouninik M, Application of the strain energy to estimate the rock load in non-squeezing ground condition, Arch Min Sci 56:551–566 (2011) Dwivedi RD, Singh M, Prediction of tunnel deformation in squeezing grounds, Eng Geol 161: 55–64 (2013) Hoek E, Big tunnels in bad rock, J Geotech Geoenviron Eng 127(9): 726–740 (2001) Mhanna M, Hussein HH, Analysis of squeezing‐induced failure in a water tunnel and measure of rehabilitation: a case study of Tishreen Tunnel, Syria, Deep Undergr Sci Eng 2024: 1–13 (2024) Sharma S, Muthreja IL, Yerpude RR, Application and comparison of squeezing estimation methods for Himalayan tunnels, Bull Eng Geol Environ 79: 205–223 (2019) Singh M, Singh B, Choudhari J, Critical strain and squeezing of rock mass in tunnels, Tunn Undergr Space Technol 22: 343–350 (2006) Singh B, Jethwa JL, Dube AK, Singh B, Correlation between observed support pressure and rock mass quality, Tunn Undergr Space Technol 7: 59–74 (1992)
Data accessibility	Data provided in this article and supplementary material in Mendeley Data. DOI: https://doi.org/10.17632/w3yhkp6zxn.1
Related research article	None

## 1 Value of the Data

• Tunnel convergence monitoring data (tunnel strains) were collected and classified into squeezing classifications according to Hoek and Marinos (2000). These data were used to evaluate the exponential graph from ground reaction curve (GRC) analysis to determine the ratio between the support pressure and the in-situ stress (*p_i_/p_o_*).
• This empirical-analytical study by GRC analysis represents all rock conditions, conducted with Geological Strength Index (*GSI*) variations ranging from 20 to 90 (very poor to excellent) for tunnel constructions at depths of 100–1000 mbgl. These GSI variations generate elastic modulus values, which influence the strain variations. Tunnel depth provides variations in material in-situ stress values, impacting the stability factor (*σ_cm_/p_o_*).
• The GRC analysis dataset can be utilized to better understand rock mass instability mechanisms during deep tunnel construction.
• This dataset is valuable for researchers and engineers in studying support capacity design for tunnels that indicate squeezing potential. They only need to collect two primary variables from the field: (1) tunnel convergence data in the form of strain (*%ε*) and (2) the stability factor (*σ_cm_/p_o_*).

## 2 Background

Squeezing is one of the instability phenomena in tunnel construction. It is a time-dependent deformation of rock that occurs around the perimeter of the opening and is usually associated with creep caused by exceeding the limiting shear stress of rock [12]. The identification and quantification of squeezing phenomena are generally conducted using empirical or semi-empirical approaches.

Previously, Hoek (1999) published a detailed analysis showing that the ratio of uniaxial compressive strength of the rock mass (*σ_cm_*) to in-situ stress (*p_o_*) can be used as an indicator of potential tunnel squeezing issues [13]. Following Sakurai’s (1983) recommendation, an analysis was conducted to determine the relationship between (*σ_cm_/p_o_*) and tunnel strain percentage [11]. Later, Hoek and Marinos (2000) expanded this analysis by incorporating internal pressure (*p_i_*) to simulate the effect of support pressure [9]. Building upon previous research, actual tunnel strain data and stability factors (*σ_cm_/p_o_*) were collected as a study of tunnel squeezing criteria, alongside ground reaction curve (GRC) calculations.

Under undisturbed conditions, the rock mass possesses its own strength, known as in-situ stress (*p_o_*). During tunnel excavation, the boundary strength—referred to as internal pressure (*p_i_*), acts radially outward from the tunnel wall and gradually decreases until maximum deformation is reached. The ratio of internal pressure to in-situ stress (*p_i_/p_o_*) is utilized as a key parameter in this study.

Furthermore, this study incorporates *GSI* values ranging from 20 to 90 in GRC calculations, representing the range of rock mass conditions as an improvement over the study by Hoek and Marinos (2000), which was limited to *GSI* 10-30.

## 3 Data Description

The compressed file in the dataset consists of a folder of GRC analytical calculations in a file titled Dataset GRC.xlsx. This Excel spreadsheet is a compilation of data on tunnel strains from the literature, processed calculations of GRC, and *p_i_/p_o_* graphs generated from the GRC calculation.

In the GRC calculation sheet, the GRC computation is outlined to determine *p_i_/p_o_*, strain (*%ε*), and the stability factor (*σ_cm_/p_o_*). The GRC incorporates the influence of rock mass strength on underground construction. The analytical process was conducted for an intact UCS (*σ_ci_*) of 30 MPa, which yields varying cohesion (*C_rm_*) and internal friction angle (*ϕ_rm_*) values across a *GSI* range of 20-90 [Table 1]. The tunnel geometry is analyzed for radii (*a*) = 5 m, 4 m, and 3 m, with an overburden thickness (*H*) ranging from 100 to 1000 m below ground level (mbgl). A similar approach is applied to the calculations using an intact UCS (*σ_ci_*) of 50 MPa. The material attributes are based on the Mohr-Coulomb Duncan-Fama technique [10], which is explained in detail in the GRC Calculation Sheet.

**Table 1:** Material properties of rock.

*σ_ci_* (MPa)	*γ* (MN/m^3^)	*Ν*	Rockmass Parameters	*GSI*							
				20	30	40	50	60	70	80	90
30	0.025	*0.2*	*C_rm_* (MPa)	0.57	0.74	0.9	1.1	1.4	1.75	2.4	3.8
			*ϕ_rm_* (°)	21	24	27	30	33	35	37.5	39
			*E_rm_* (MPa)	670	1640	3980	9340	20360	38830	61170	79635
			*K*	2.12	2.37	2.66	3.00	3.39	3.69	4.11	4.40
			*σ_cm_* (MPa)	1.66	2.28	2.94	3.81	5.16	6.72	9.73	15.93
50	0.025	*0.2*	*C_rm_* (MPa)	0.75	0.98	1.20	1.46	1.82	2.43	3.58	5.89
			*ϕ_rm_* (°)	23	27	30	33	36	39	41	42
			*E_rm_* (MPa)	670	1640	3980	9340	20360	38830	61170	79635
			*K*	2.31	2.66	3.02	3.42	3.87	4.33	4.76	5.06
			*σ_cm_* (MPa)	2.29	3.19	4.17	5.39	7.15	10.10	15.64	26.50

*Note: σ_ci_* = UCS intact; *γ* = Rock density; ν = Poisson’s Ratio; *GSI* = Geological Strength Index; *C* = Rock mass cohesion; *ϕ =* Rock mass internal friction angle; *E_rm_* = Modulus elasticity of rock mass; *K* = Slope from Mohr envelope; *σ_cm_* = UCS of Rock mass *H* = Tunnel depth from surface; *p_o_* = In-situ stress.

**Fig. 1:**
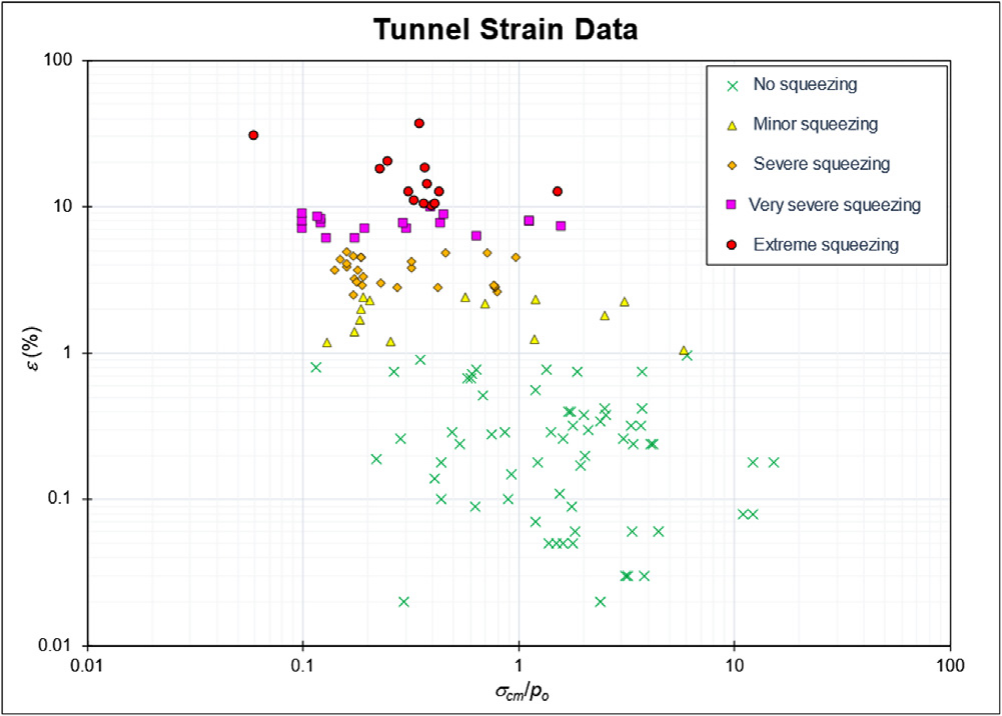
Distribution of tunnel strains from 135 tunnels that experienced squeezing and non-squeezing conditions.

The actual dataset from the tunnel wall convergence monitoring comprises 135 strain data points collected from previous studies on tunnels located in Nepal, India, Iran, Turkey, China, Japan, and Italy (see Data Strain Literature sheet) [[1], [2], [3], [4], [5], [6], [7], [8]]. The dataset in each column contains: Number (No), Name of tunnel, References, Location, Rock Type, Q, RMR, m_i_, *GSI*, Density (*ɣ*), Tunnel radius (*a*), Overburden height (*H*), UCS Intact *σ_ci_*, UCS Rock mass *σ_rm_, p_o_* (In-situ stress), Tunnel strain (*%ε*), Deformation modulus of rock mass (*E_rm_*), Deformation modulus of intact rock (*E_i_*), Rock stability (*σ_cm_/p_o_*), Squeezing category, and Literature Sources. These tunnels are intended for both civil construction and underground mining purposes, with an average depth of 300 mbgl, an average tunnel radius (*a*) of 4 m, and a rock density (*γ*) of 2.6 t/m³. The rock lithologies, which include schist, dolomite, limestone, gneiss, phyllite, clay, sandstone, and shale, vary as a result of the tunnel’s locations. According to lithological analysis, strain that has >1 % convergence in tunnels found in phyllites and clay formations can range from 1 % to 36.7 %. These data were classified using Hoek and Marinos’ squeezing classification [9] based on tunnel strains (Fig. 1). The classification of squeezing percentage is presented in Table 2.

**Table 2:** Squeezing classification by Hoek (2000).

No	Squeezing classification	Strain (%)
1	No Squeezing	0–1
2	Minor Squeezing	1–2.5
3	Severe Squeezing	2.5–5
4	Very Severe Squeezing	5–10
5	Extreme Squeezing	10–100

### 3.1 Analytical calculation

**Fig. 2:**
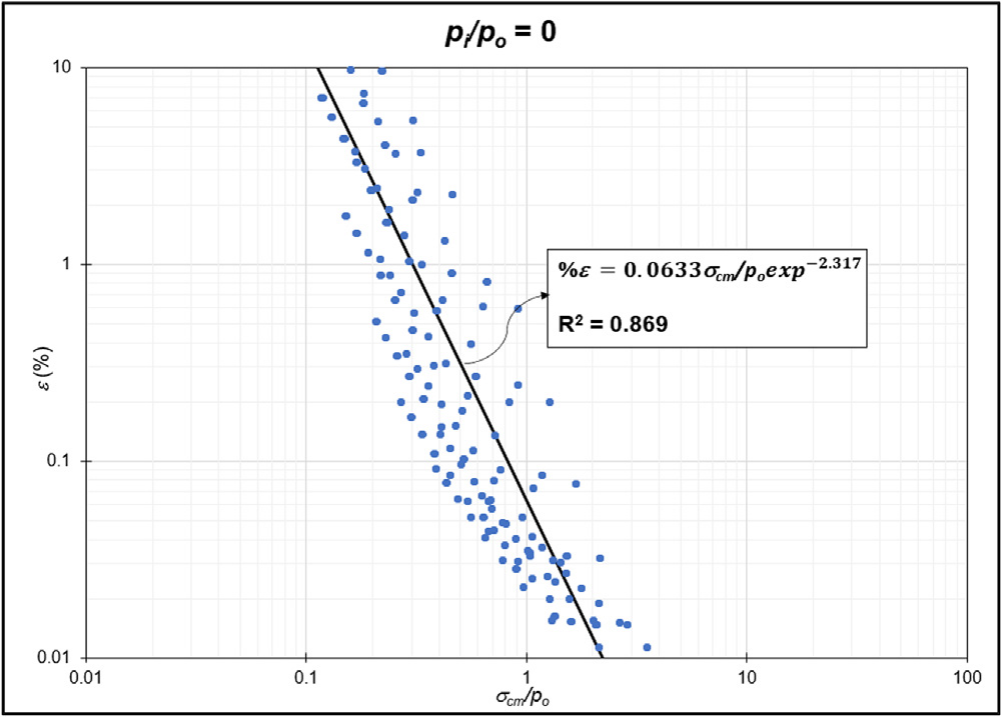
Regression of stability factor vs. strain for *p_i_/p_o_* = 0.

**Fig. 3:**
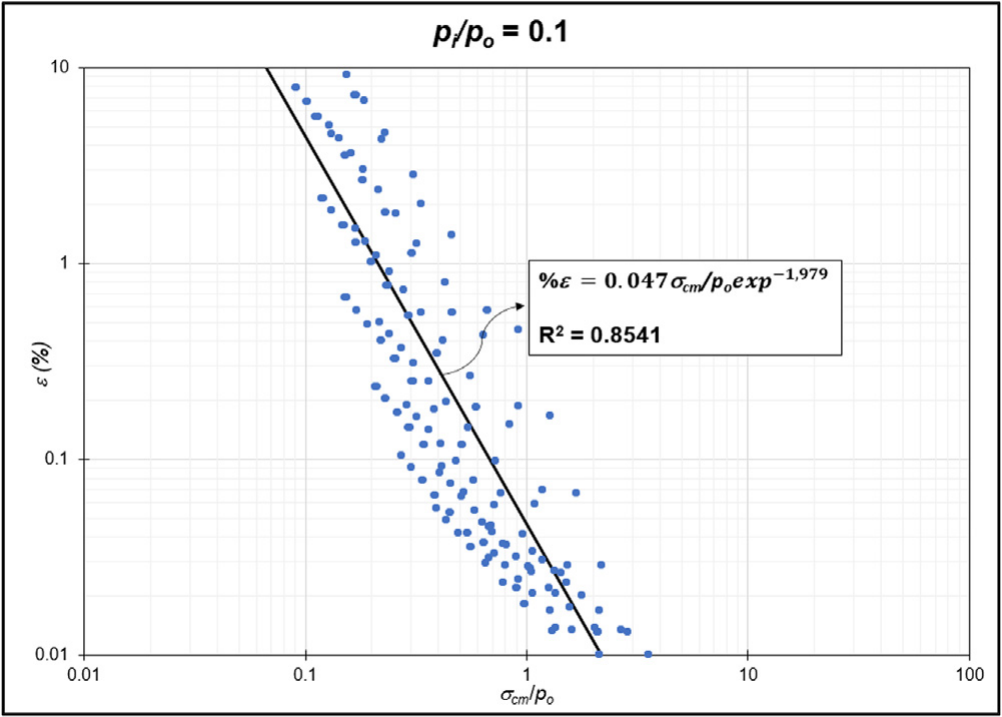
Regression of stability factor vs. strain for *p_i_/p_o_* = 0.1.

**Fig. 4:**
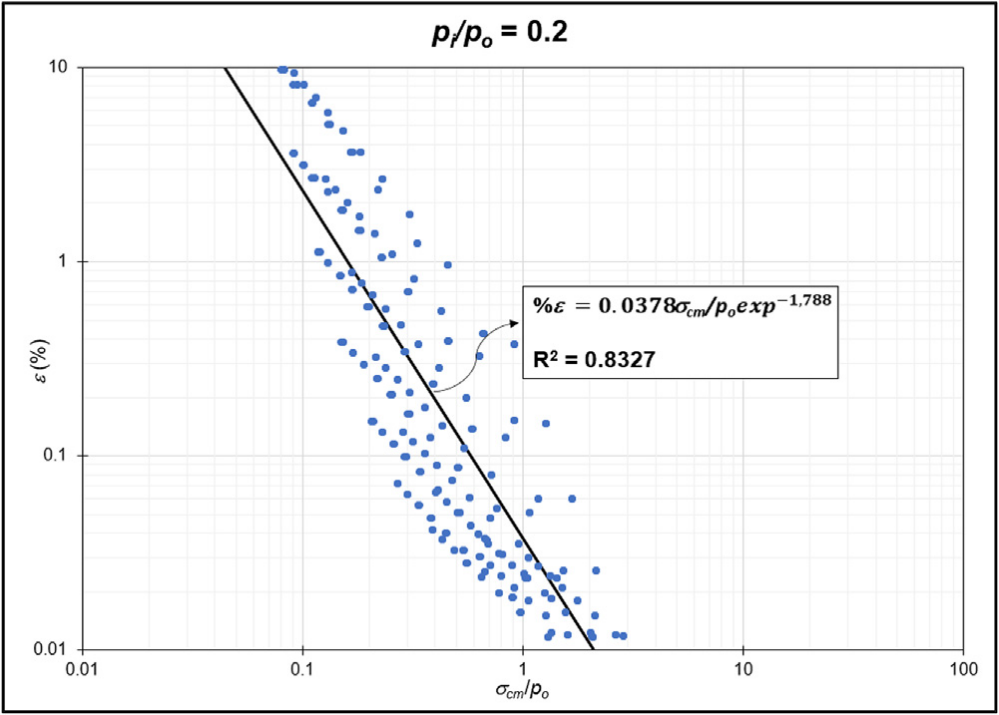
Regression of stability factor vs. strain for *p_i_/p_o_* = 0.2.

**Fig. 5:**
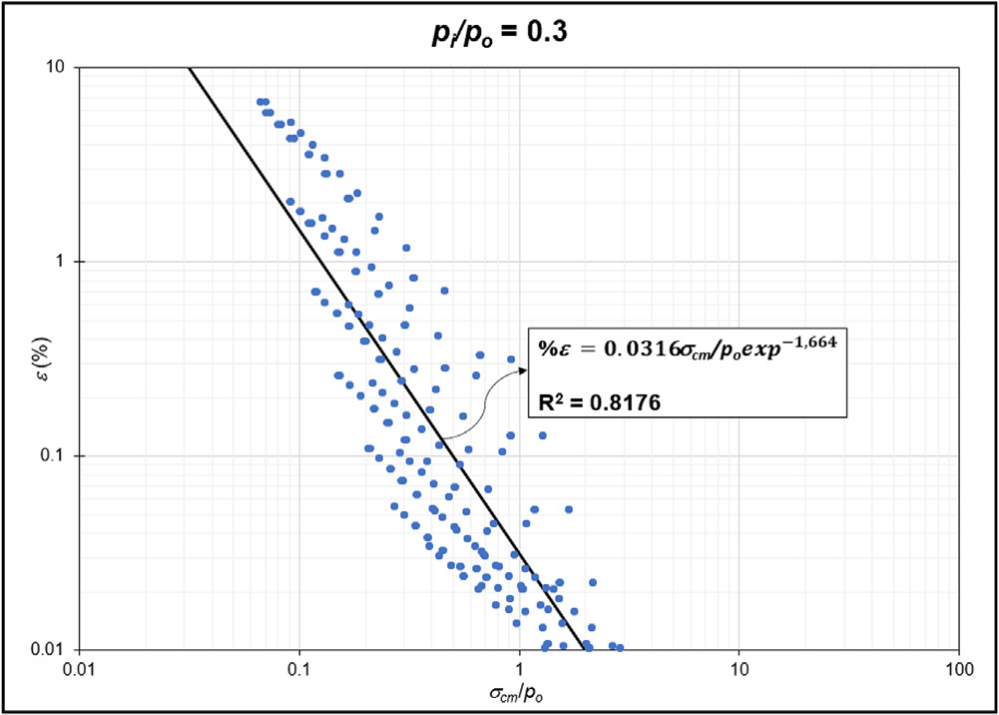
Regression of stability factor vs. strain for *p_i_/p_o_* = 0.3.

**Fig. 6:**
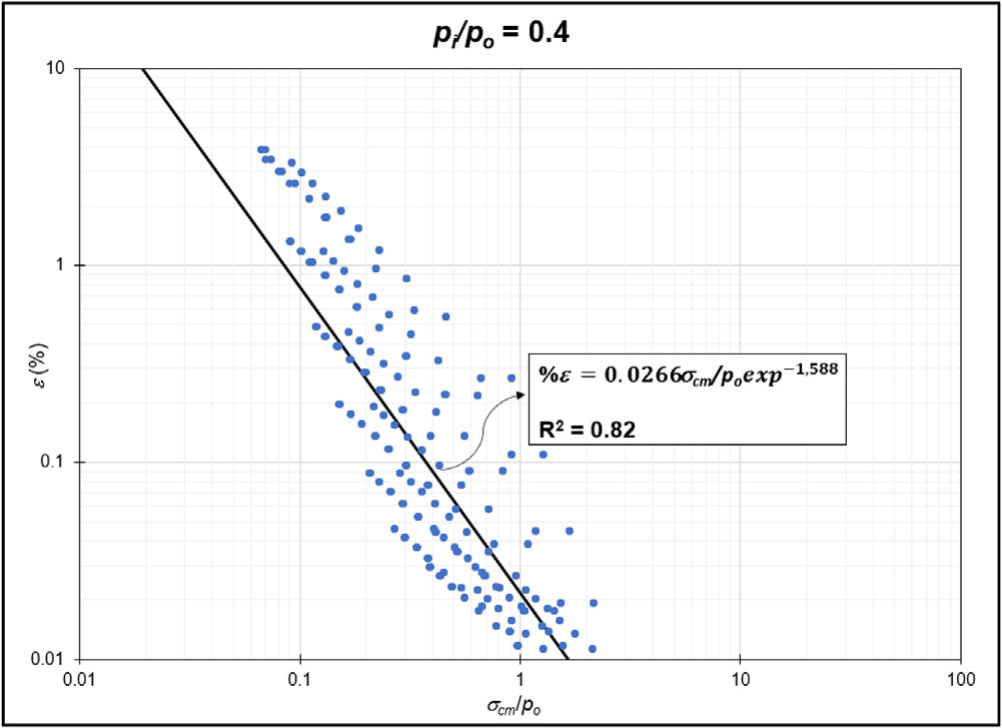
Regression of stability factor vs. strain for *p_i_/p_o_* = 0.4.

**Fig. 7:**
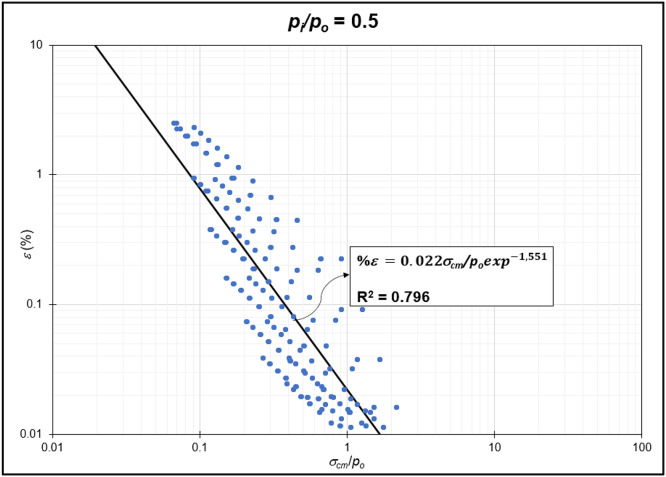
Regression of stability factor vs. strain for *p_i_/p_o_* = 0.5.

**Fig. 8:**
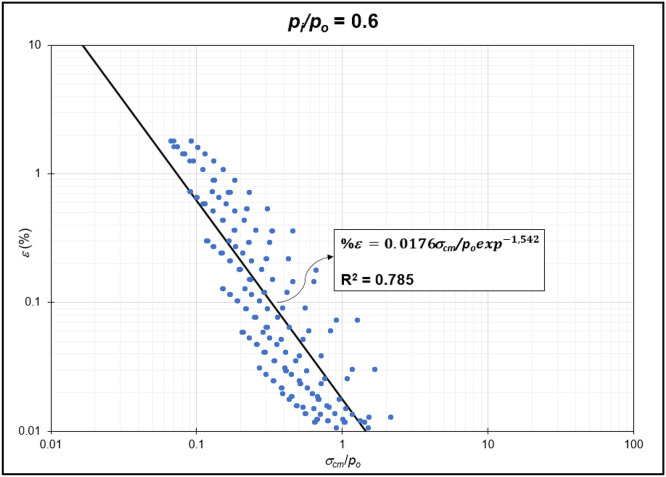
Regression of stability factor vs. strain for *p_i_/p_o_* = 0.6.

**Fig. 9:**
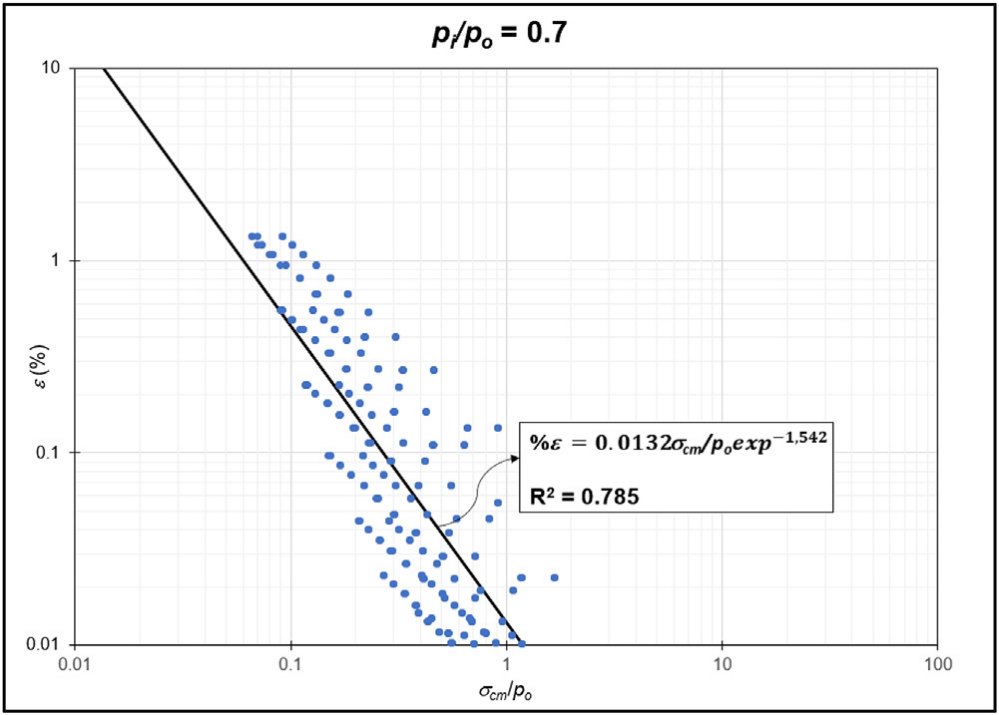
Regression of stability factor vs. strain for *p_i_/p_o_* = 0.7.

**Fig. 10:**
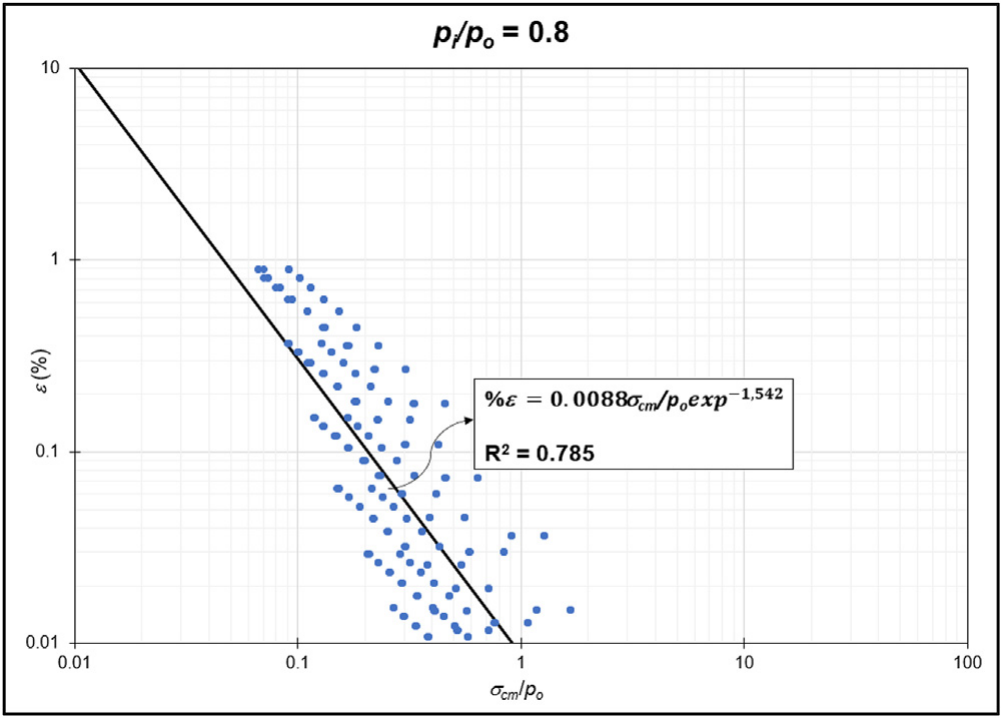
Regression of stability factor vs. strain for *p_i_/p_o_* = 0.8.

**Fig. 11:**
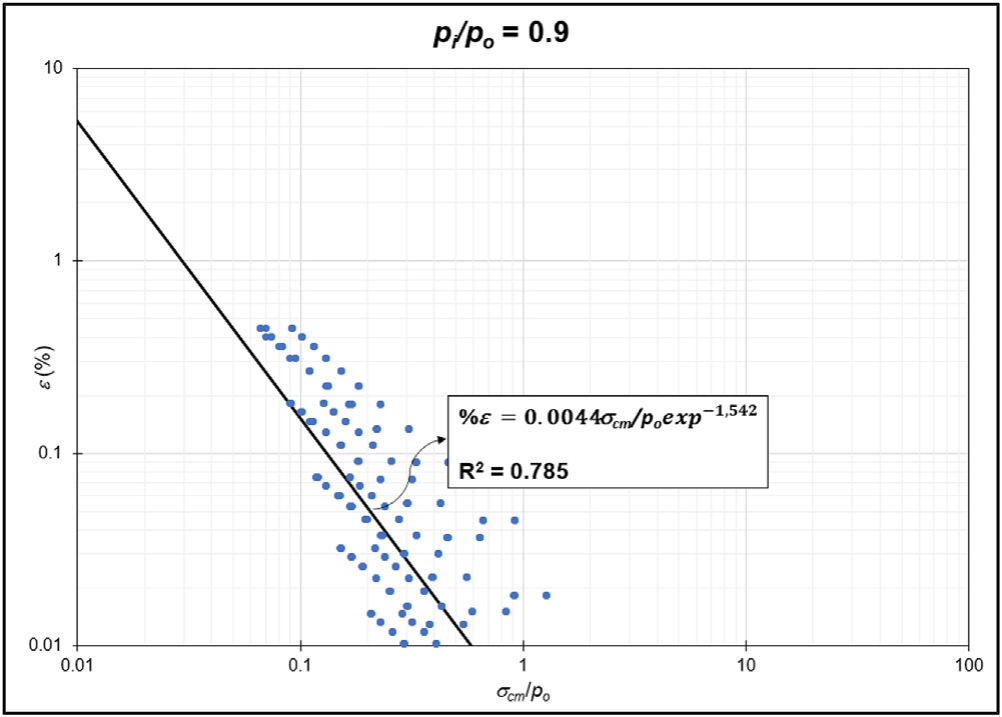
Regression of stability factor vs. strain for *p_i_/p_o_* = 0.9.

The GRC calculation concept based on the Mohr-Coulomb failure criterion [10] was used to generate the reduction of internal pressure relative to in-situ stress (*p_i_/p_o_*) from elastic to plastic conditions at maximum displacement. Since *p_i_/p_o_* = 1 represents the condition of the rock mass without disturbance (in-situ rock mass), the *p_i_/p_o_* interval was generated from 0 to 0.9. In the same Excel file, *p_i_/p_o_* from GRC calculations were classified into *p_i_/p_o_* = 0, *p_i_/p_o_* = 0.1, *p_i_/p_o_* = 0.2, *p_i_/p_o_* = 0.3, *p_i_/p_o_* = 0.4, *p_i_/p_o_* = 0.5, *p_i_/p_o_* = 0.6, *p_i_/p_o_* = 0.7, *p_i_/p_o_* = 0.8, and *p_i_/p_o_* = 0.9 for the specific strain (*%ε*) and stability factor (*σ_cm_/p_o_*) variables (Fig. 2) to (Fig. 11). The relationship between two variables can be observed through the correlation coefficient for each *p_i_/p_o_*, which was plotted in a power regression graph. It is evident that the relationship between the variables falls within the strong category (0.78–0.87), indicating that the *p_i_/p_o_* exponential graph in relation to strain (*%ε*) and the stability factor (*σ_cm_/p_o_*) is reliable for use [Table 3] (Fig. 3, Fig. 4, Fig. 5, Fig. 6, Fig. 7, Fig. 8, Fig. 9, Fig. 10).

**Table 3:** Strain GRC exponential equation.

*p_i_/p_o_*	Equation	Equation No
0	$\varepsilon =0.06\;\sigma \text{cm}/po\;exp^{-2.31}$	(1)
0.1	$\varepsilon =0.05\;\sigma \text{cm}/po\;exp^{-1.98}$	(2)
0.2	$\varepsilon =0.04\;\sigma \text{cm}/po\;exp^{-1.79}$	(3)
0.3	$\varepsilon =0.03\;\sigma \text{cm}/po\;exp^{-1.66}$	(4)
0.4	$\varepsilon =0.03\;\sigma \text{cm}/po\;exp^{-1.59}$	(5)
0.5	$\varepsilon =0.02\;\sigma \text{cm}/po\;exp^{-1.50}$	(6)
0.6	$\varepsilon =0.02\;\sigma \text{cm}/po\;exp^{-1.54}$	(7)
0.7	$\varepsilon =0.01\;\sigma \text{cm}/po\;exp^{-1.54}$	(8)
0.8	$\varepsilon =0.009\;\sigma \text{cm}/po\;exp^{-1.54}$	(9)
0.9	$\varepsilon =0.004\;\sigma \text{cm}/po\;exp^{-1.54}$	(10)

**Fig. 12:**
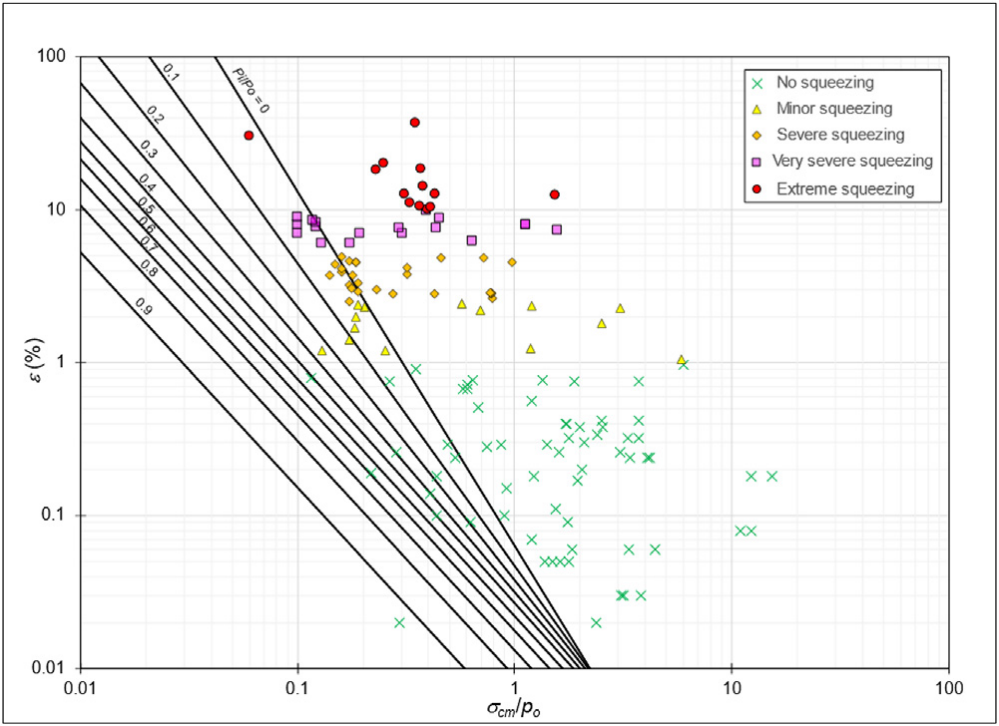
The plot of 135 tunnel strains data points, along with *p_i_/p_o_* lines derived from GRC.

The 135 tunnel strain data are plotted on the exponential graph derived from GRC data (Fig. 12). As a graph representing the support effect, the tunnel strain data indicate that tunnels experiencing the squeezing phenomenon predominantly occur when *p_i_/p_o_* < 0.1. Therefore, a minimum *p_i_/p_o_* = 0.1 is required to prevent the occurrence of squeezing conditions.

## Limitations

The analysis was conducted using the ground reaction curve method, which is limited to 2D analysis under a hydrostatic condition, and the tunnel strain data consist mainly of tunnel construction in Asia, with a few points from Europe.

## Ethics Statement

The authors have read and followed the ethical requirements for publication in *Data in Brief* and confirm that the current work does not involve human subjects, animal experiments, or any data collected from social media platforms.

## CRediT Author Statement

**Ferlien Margareth Mareyke Mombilia**: Conceptualization, Methodology, Calculation, Writing – Original draft; **Simon Heru Prassetyo**: Conceptualization, Methodology, Supervision, Writing – Review and Editing; **Yudhidya Wicaksana**: Supervision, Methodology, Data Curation; **Ridho Kresna Wattimena**: Supervision, Validation Data, Review; **Anatasya Claresta**: Collecting Data, Review and Editing

## Acknowledgments

The authors acknowledge funding from the ITB Excellent Research Program 2024, grant number 959/IT1.B07.1/TA.00/2024.

## Declaration of Competing Interest

The authors declare that they have no known competing financial interests or personal relationships that could have appeared to influence the work reported in this paper.

## Data Availability

Mendeley DataDataset GRC.xlsx (Original data).
